# Mediterranean diet versus low-fat diet on cardiovascular disease (CVD) risk factors and outcomes: A systematic review of RCTs

**DOI:** 10.1097/MD.0000000000047971

**Published:** 2026-03-13

**Authors:** Maneeth Mylavarapu, Aarushi Batra, Israel Garcia, Abirami Balasubramanian, Muhammad Ammar Husnain, Ragha Harshitha Atla, Poorna Sai Harshini Muttuluru, Elias Abboud, Alisson Silva

**Affiliations:** a Department of Cardiology, Endeavor Health Cardiovascular Institute, Endeavor Health Glenbrook Hospital, Glenview, IL; b Division of Cardiology, Department of Medicine, University of Chicago Pritzker School of Medicine, Chicago, IL; c Department of Internal Medicine, LLRM Medical College, Meerut, India; d Department of Internal Medicine, St. George’s University School of Medicine, True Blue, Grenada; e Department of Internal Medicine, Stanley Hospital and Medical College, Chennai, India; f Department of Internal Medicine, CMH Lahore Medical College, Lahore, Pakistan; g Department of Internal Medicine, Bicol Christian College of Medicine, Legazpi City, Philippines; h Department of Internal Medicine, Government Medical College, Anantapur, India; i Department of Internal Medicine, Faculty of Medicine, Saint Joseph University, Beirut, Lebanon; j Department of Critical Care Medicine, UNIME University School of Medicine, Tereza de Lisieux Hospital, Lauro de Freitas, Brazil.

**Keywords:** cardiovascular risk factors, dietary inflammatory index, low-fat diet, Mediterranean diet, randomized control trials, systematic review

## Abstract

**Background:**

The Mediterranean diet (MD) and low-fat diet (LFD) were well-known dietary interventions for cardiovascular disease prevention. However, there is an ongoing debate about the relative efficacy of improving cardiovascular (CV) risk factors and clinical outcomes between the 2. This systematic review aims to evaluate and summarize findings from randomized controlled trials (RCTs) that directly compare the MD and LFD concerning CV risk factors and clinical outcomes.

**Methods:**

Per the Preferred Reporting Items for Systematic Reviews and Meta-analyses guidelines, a comprehensive literature search was conducted across PubMed, Google Scholar, SCOPUS, Science Direct, and RCTs that compared MD and LFD in adults, and reported data on CV risk factors or clinical outcomes were included.

**Results:**

Our systematic review includes 11 RCTs with 5942 participants. MD significantly reduced the formation of procoagulant and prothrombotic microvesicles. Studies have also reported that MD has resulted in greater reductions in total cholesterol, cholesterol-high-density lipoproteins ratios, insulin levels, blood glucose levels, leukocyte count, and body mass index compared to LFD.

**Conclusion:**

MD demonstrates superior efficacy over LFD in improving CV risk factors and outcomes. These findings could support the preferential recommendation of MD over LFD for the secondary prevention of cardiovascular diseases. Further research should explore the long-term adherence and implementation of these dietary interventions in diverse populations.

## Graphical abstract

**Figure:**
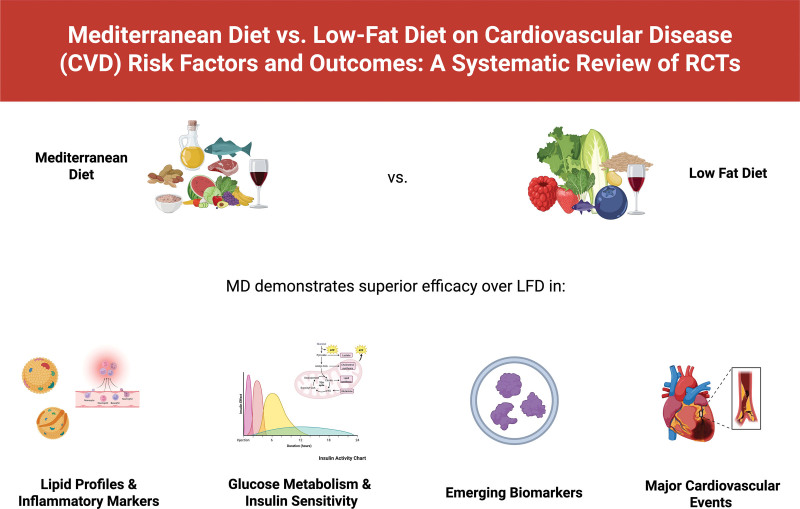

## 1. Introduction

Cardiovascular diseases (CVDs) continue to remain the leading cause of mortality globally, with their prevalence steadily increasing.^[1,2]^ Almost one-third of global deaths in 2021 (20.5 million deaths) were due to CVDs, and it’s projected to rise to 23 million by 2030.^[3,4]^ Of the existing burden, over 75% occurs in low- and middle-income countries (low-income countries and middle-income countries), where access to preventive healthcare and treatment options is often limited.^[5,6]^ World Health Mission 2030 seeks a collaborative strategy to significantly reduce this figure and transform cardiovascular (CV) health.^[7]^ Recognizing that as many as 80% of early CV deaths and strokes are preventable, the mission emphasizes prioritizing the prevention of CVD.^[7-9]^

Modifiable risk factors such as dyslipidemia, hypertension, insulin resistance, obesity, and systemic inflammation play a central role in the pathogenesis of CVD, and diet is a key determinant of many of these risk markers.^[10]^ The collective approach by World Health Mission 2030 places a strong emphasis on modifiable risk factors that vary across different populations, regions, and lifestyles. These factors include promoting regular physical exercise, adopting healthy dietary habits, encouraging tobacco cessation, and mitigating air pollution.^[7,11]^ Multiple studies have proven that physical activity and changes in dietary habits have a more significant impact on reducing CVD symptoms and disease burden.^[8,12,13]^ Notably, greater adherence to a nutritional diet has decreased the risk of CVD mortality by at least 12% to 28%, as evidenced by Reedy et al.^[14]^ Diets rich in foods with anti-inflammatory properties, including fruits, vegetables, nuts, and legumes, slow down atherosclerosis, a major contributor to CVD.^[15,16]^ Furthermore, studies have reported that the risk of developing CVD increases by 6% for every 1g rise in dietary sodium intake.^[17]^

Two major diets that have been proven beneficial for CV health are the Mediterranean diet (MD) and the low-fat diet (LFD). The Mediterranean diet is a traditional diet distinguished by abundant fruits, vegetables, monounsaturated fats such as olive oil, fish, whole grains, legumes, and nuts, and low consumption of saturated fats, meat, and dairy products.^[18,19]^ It also incorporates a moderate intake of alcohol in the form of red wine due to the presence of bioactive polyphenols, attributing anti-inflammatory properties.^[19]^ This particular dietary pattern decreases the expression of proatherogenic genes and levels of proinflammatory markers.^[15,20]^ On the other hand, LFD restricts calorie intake from fats to no more than 30%, minimizing intake of saturated fats to <7% of total calories and cholesterol to <200 mg/day, thereby facilitating significant weight reduction, ideal for the rising obesity rate.^[21]^ Adopting a low-fat diet helps prevent the incidence and recurrence of CVDs by reducing circulating cholesterol levels.^[21,22]^

Both the MD and LFD have positive impacts on CV health. Despite the common belief that lower fat content equates to healthier dishes, the low-fat diet, as its name suggests, may not necessarily be superior to the Mediterranean diet.^[23]^ Furthermore, recommendations for both these diets may not be universally suitable for all populations owing to differences in culture, economic status, and regional food availability.^[24]^ Hence, further research and trials were necessary to educate individuals on the nutritional benefits and effectiveness of these diets in preventing CV events before providing counseling or recommendations.^[25]^ Several randomized controlled trials (RCTs) have evaluated the impact of these 2 dietary patterns on CV risk factors and clinical outcomes. While individual trials suggest that the MD may offer superior benefits in terms of many CV risk factors, the evidence continues to remain scattered and heterogeneous. Given the growing interest in personalized nutrition and evidence-based dietary guidelines, a systematic synthesis of the current evidence is warranted to clarify the comparative effectiveness of Mediterranean versus low-fat diets. This systematic review aims to evaluate and summarize findings from randomized controlled trials that directly compare the MD and LFDs with respect to CV risk factors and clinical outcomes. By consolidating the existing data, this review seeks to inform clinical practice and public health recommendations for dietary strategies in the prevention and management of CVD.

## 2. Materials and methods

**Figure 1.**
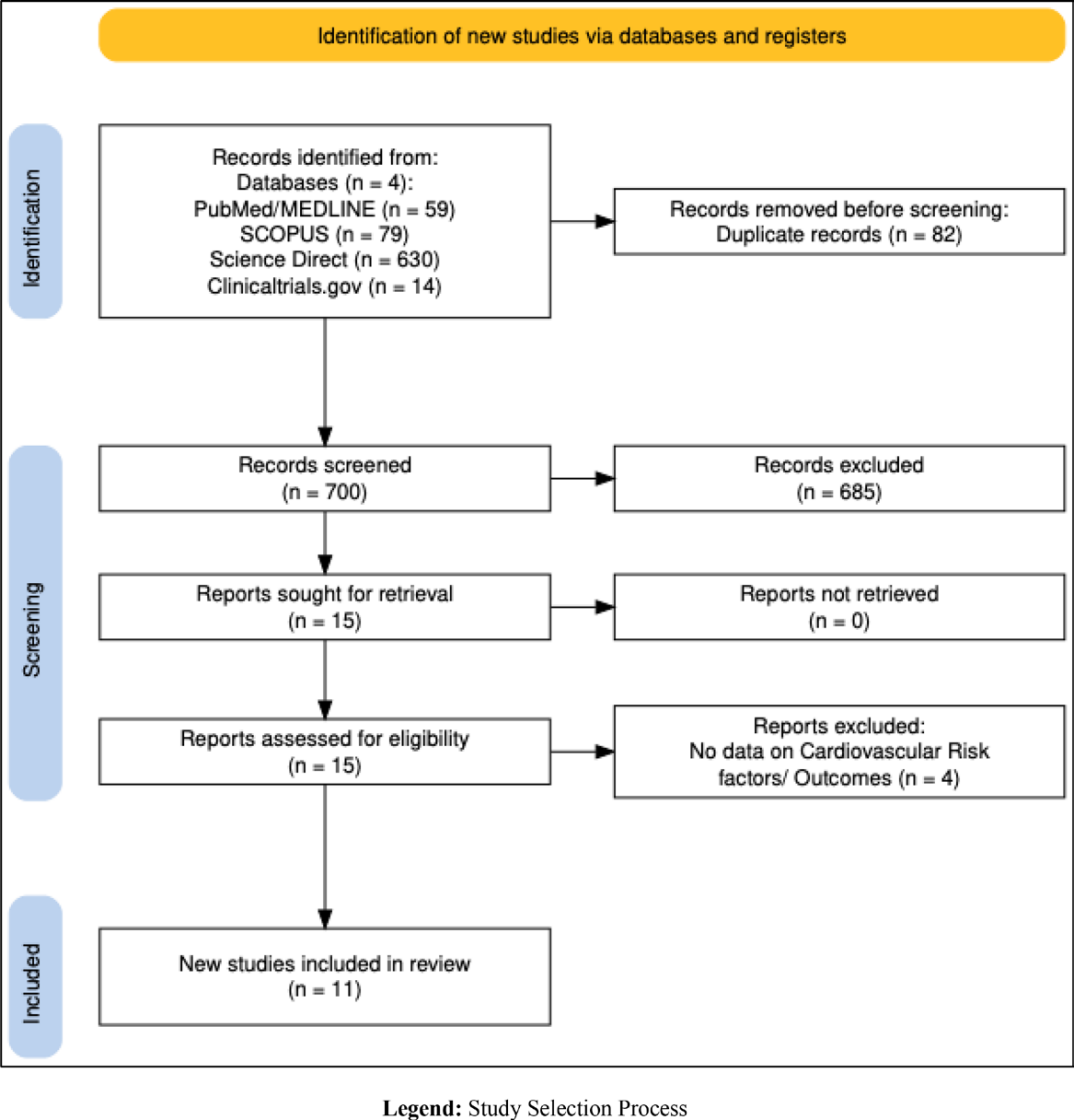
PRISMA flow chart of study selection process. PRISMA = Preferred Reporting Items for Systematic Reviews and Meta-analyses

**Figure 2.**
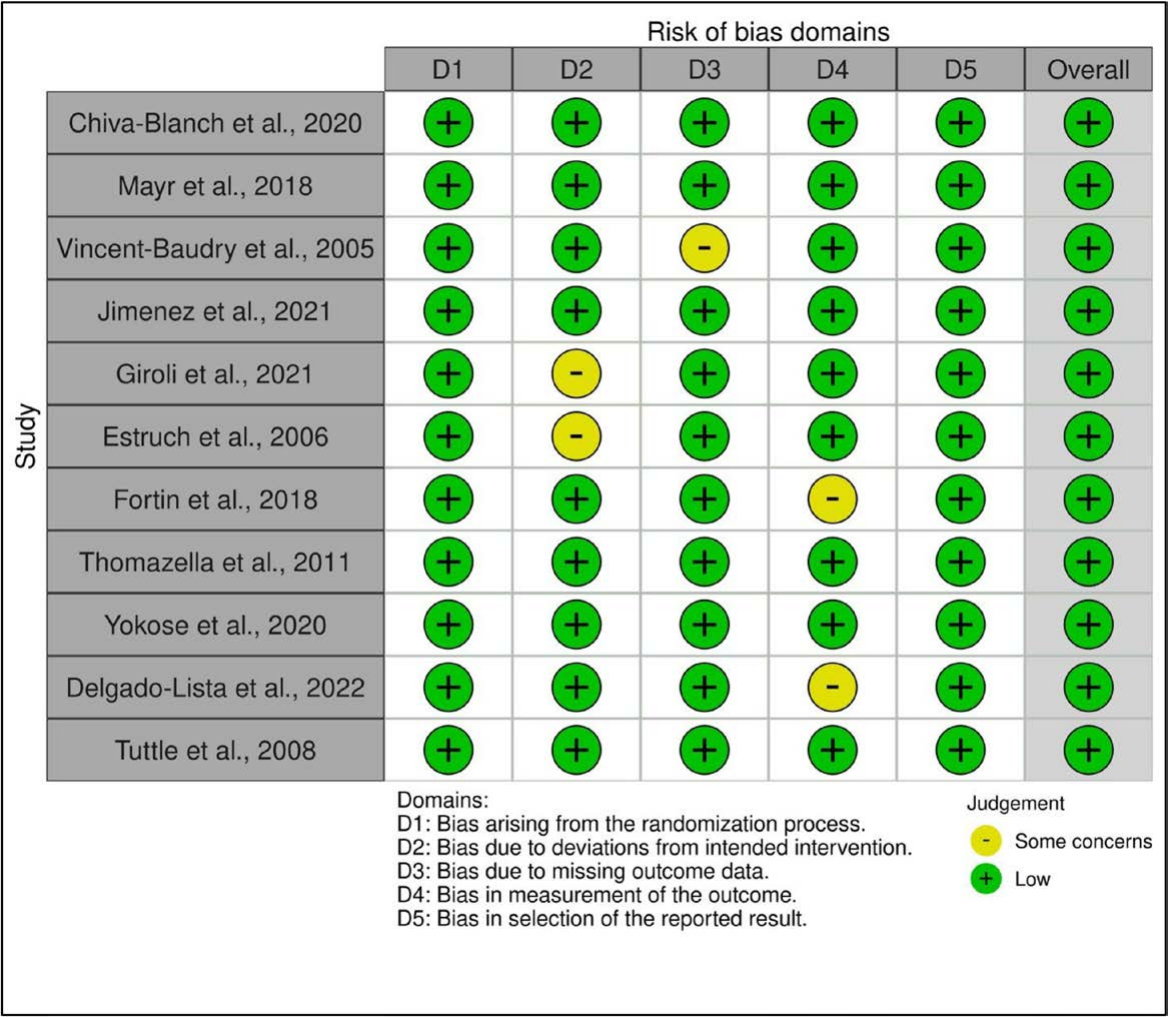
Risk of bias assessment of included studies.

Per the Preferred Reporting Items for Systematic Reviews and Meta-analyses guidelines,^[26]^ a comprehensive literature search was conducted in several prominent, reliable databases, including PubMed/MEDLINE, Scopus, clinicaltrials.gov, and Science Direct. Subject headings and keywords related to “Mediterranean diet,” “low-fat diet,” “cardiovascular disease,” “CVD,” “cardiovascular risk factors,” and “cardiovascular events” were included. The references of the selected studies were also examined to verify the comprehensiveness of the search. The resulting search strategy utilized to obtain the necessary studies for this paper is outlined in Supplementary File S1, Supplemental Digital Content, https://links.lww.com/MD/R504. RCTs comparing the impact of MD and LFDs on CVD risk factors and CV events are included in the study. A detailed list of inclusion and exclusion criteria is outlined in Supplementary File S2, Supplemental Digital Content, https://links.lww.com/MD/R504. The study protocol was registered in the PROSPERO database (International Prospective Register of Systematic Reviews) ID: CRD42024517761. Two author participants, IG and MH, completed the screening of titles and abstracts independently, while the independent full-text review was completed by 2 different reviewers, AB and AS. The third reviewer, MM, resolved conflicts concerning the screening. Figure 1 outlines the study selection process.^[27]^ The risk of bias (RoB) assessment regarding the methodological quality of the included studies was conducted separately (blinded) by 2 reviewers, MA and RH, using RoB 2.0^[28]^ and visualized with Robvis software (Fig. 2).^[29]^ This study was based on a systematic review of previously published articles, so no ethical approval was required. All included studies were assumed to have appropriate ethical approval.

## 3. Results

Our systematic review included 11 RCTs^[30-40]^ with 5942 participants. The included studies had varied follow-ups, ranging from 3 months to 7 years, dietary compositions, and outcome assessments. The MD interventions frequently incorporated supplemental olive oil or nuts, while the LFD group consisted of traditional low-fat dietary recommendations.

### 3.1. Cardiovascular risk factors

Nine studies^[30-38]^ reported on traditional and emerging CV risk factors. Across these studies, MD consistently demonstrated favorable effects on lipid profiles, inflammatory markers, blood pressure, and insulin sensitivity. Table 1 outlines key findings comparing MD and LFD on CV risk factors.

**Table 1:** Key findings on cardiovascular (CV) risk factors.

Author(s), year	Population characteristics	Total patients	MD	LFD	Follow-up duration	Outcome measures	Key findings
Chiva-Blanch et al, 2020	Patients at risk of CVD, assessing microvesicle levels	106 (MD = 53; LFD = 53)	MD + olive oil or nuts	LFD	1 yr	Circulating microvesicles (cMV), procoagulant and prothrombotic markers	MD significantly reduced prothrombotic cMV (*P* < .0001). LFD reduced proinflammatory cMV more (*P* = .038)
Mayr et al, 2018	Adults with or at risk of CHD	56 (MD = 27; LFD = 29)	MD	LFD	6 mo	Dietary Inflammatory Index (DII), CRP, IL-6	MD had greater reduction in DII (*P* = .008), but no significant changes in CRP or IL-6
Vincent-Baudry et al, 2005	Adults with elevated metabolic risk	169 (MD = 88; LFD = 81)	MD	LFD	3 mo	BMI, total cholesterol, triacylglycerols, insulin levels	MD significantly reduced cholesterol, triglycerides, and insulin vs LFD (*P* < .05). Both improved BMI
Jimenez et al, 2021	Adults with CHD or atherosclerosis risk	731 (MD = 396; LFD = 335)	MD	LFD	5 and 7 yr	IMT-CC, carotid plaque height and number	MD reduced IMT-CC and plaque height significantly (*P* < .001); no change in plaque number
Giroli et al, 2021	Patients postcoronary stenting	130 (MD = 64; LFD = 66)	MD	LFD	3 mo	Blood fatty acid profile	MD improved omega-3 and n − 6/n − 3 ratio more than low-fat. Saturated fat decreased in both
Estruch et al, 2006	Adults at high CV risk		MD + olive oil or nuts	LFD	4.8 yr	BP, glucose, cholesterol-HDL ratio, CRP, IL-6, ICAM-1, VCAM-1	MD improved BP, glucose, lipids, insulin, and inflammatory markers more than low-fat diet
Fortin et al, 2018	Overweight adults	28 (MD = 14; LFD = 14)	MD	LFD	6 mo	Waist circumference, BMI, HDL	Both diets reduced BMI and waist circumference. HDL improved in women on Mediterranean diet
Thomazella et al, 2011	Adults with CHD risk	40 (MD = 21; LFD = 19)	MD	TLCD (low-carbohydrate diet)	12 wk	Total/LDL cholesterol, oxidized LDL, leukocyte count, HDL	TLCD reduced LDL more. MD lowered leukocyte count (*P* = .025); improved monounsaturated fat intake
Yokose et al, 2020	Adults with overweight/obesity	161 (MD = 76; LFD = 85)	MD	LFD	6 and 24 mo	Weight, lipid profile, SBP, fasting insulin	Both diets improved weight, HDL, cholesterol/HDL ratio, and insulin. MD had more sustained effects at 24 mo

BMI = body mass index, CHD = coronary heart disease, cMV = circulating microvesicles, CRP = C-reactive protein, CV = cardiovascular, CVD = cardiovascular disease, DII = dietary inflammatory index, HDL = high-density lipoprotein, ICAM-1 = intercellular adhesion molecule-1, IL-6 = interleukin-6, IMT-CC = intima-media thickness of the common carotid artery, LDL = low-density lipoprotein, LFD = low-fat diet, MD = Mediterranean diet, TLCD = Total low carbohydrate diet, VCAM-1 = vascular cell adhesion molecule 1.

#### 3.1.1. Lipid profiles and inflammatory markers

Studies reported significant improvements in lipid parameters with the MD. Vincent-Baudry et al^[32]^ observed significantly greater reductions in total cholesterol and triacylglycerols with the MD compared to LFD (*P* < .05), while Giroli et al^[34]^ demonstrated improved omega-3 fatty acid levels and a reduced n − 6/n − 3 ratio in the MD group. Estruch et al^[35]^ showed that the MD significantly decreased total cholesterol, improved high-density lipoproteins levels, and reduced inflammatory markers, including C-reactive protein, interleukin-6, intercellular adhesion molecule-1, and vascular cell adhesion molecule 1 (VCAM-1).

#### 3.1.2. Glucose metabolism and insulin sensitivity

Both Estruch et al^[35]^ and Yokose et al^[38]^ found that the MD led to improved fasting insulin concentrations and glycemic control, with sustained effects observed over 24 months in glycemic control.

#### 3.1.3. Blood pressure and anthropometric indices

Studies have reported that MD significantly reduces blood pressure. As evidenced by Estruch et al^[35]^ and Yokose et al,^[38]^ the MD diet group reported a significant drop in both systolic and diastolic blood pressures. Although both MD and LFD had improved effects on body weight and waist circumference, the MD group had better dietary adherence and slightly greater reductions in body mass index, as reported by Fortin et al^[36]^ and Vincent-Baudry et al^[32]^

#### 3.1.4. Emerging biomarkers

Chiva-Blanch et al^[30]^ demonstrated that the MD significantly reduced circulating prothrombotic and procoagulant microvesicles (*P* < .0001), while Mayr et al^[31]^ showed greater anti-inflammatory dietary index improvement with the MD, though without corresponding changes in C-reactive protein or interleukin-6.

### 3.2. Cardiovascular outcomes

Two studies, Delgado-Lista et al^[39]^ and Tuttle et al,^[40]^ specifically addressed CV outcomes, including myocardial infarction, stroke, heart failure, cardiac death, and the need for revascularization procedures. Delgado-Lista et al^[39]^ reported that the MD significantly reduced major CV events compared to the LFD in patients with established coronary artery disease over a median follow-up of 7 years (87 vs 111 events; hazard ratio = 0.719; *P* = .024). Tuttle et al^[40]^ found that both the Mediterranean and low-fat diets significantly reduced composite CV events compared to usual care (*P* < .001); however, no significant difference was observed between the 2 diet groups. Table 2 outlines key findings of comparing MD and LFD on CV outcomes.

**Table 2:** Key findings on cardiovascular (CV) outcomes.

Author(s), year	Population characteristics	Intervention	Comparator	Follow-up duration	Outcome measures	Key findings
Delgado-Lista et al, 2022	1002 patients with diagnosed CHD	MD	LFD	7 yr	Major CV events, lipid profile	Mediterranean diet reduced primary CV events (87 vs 111, HR = 0.719, *P* = .024), especially in men
Tuttle et al, 2008	Post-MI patients	MD	LFD + usual care	46 mo	Composite CV events (MI, angina, HF, stroke, cardiac deaths)	Both diets reduced events vs control (*P* < .001). No significant difference between Mediterranean and low-fat diet groups

CHD = coronary heart disease, CV = cardiovascular, HF = heart failure, HR = hazards ratios, LFD = low-fat diet, MD = Mediterranean diet, MI = myocardial infarction.

## 4. Discussion

Our systematic review, comparing the MD and LFD on CV health, aligns with the growing body of evidence suggesting the superiority of the MD, particularly concerning CV risk factors.^[41-44]^ Our findings report that the MD reduced the progression of atherosclerosis due to improved lipid profiles and glucose metabolism and reduced the inflammatory burden due to its action on the dietary inflammatory index. Furthermore, our study findings also report that MD was superior to LFD in reducing major CV events in patients with established coronary artery disease, highlighting its role in secondary prevention. Although multiple studies recommended MD for secondary prevention of CVD,^[41,45]^ significant evidence was not found owing to the limitations in the previously available trials.^[46,47]^ However, the CORonary Diet Intervention with Olive oil and CV PREVention (CORDIOPREV) study^[39]^ finally provided the evidence required, concluding that the MD was superior to the LFD in preventing major CV events.

The benefits observed in MD compared to LFD could be explained by the fact that MD focuses on an overall nutrient-rich approach, while LFD primarily targets reduction in dietary fat intake. MD emphasizes intake of monounsaturated fats, polyphenols, and omega-3 fatty acids, which enhance lipid profiles, reduce inflammation, and improve endothelial function.^[48]^ Additionally, MD provided greater intakes of soluble fibers (from fruit, vegetables, legumes, and cereals) and monounsaturated fatty acids (oleic acid from olive oil), which are known independently to have a cholesterol- and LDL cholesterol-lowering effect.^[32]^ It also lowers systemic inflammation and promotes better glycemic control, while the LFD’s carbohydrate reliance can increase insulin resistance and inflammation over time.^[49]^ Regarding blood pressure, MD’s superior benefit may stem from the inclusion of α-linolenic acid from walnuts, which is linked to improved blood pressure levels.^[50]^ Another plausible explanation of the impact of MD on blood pressure could be the resemblance between MD and the Dietary Approaches to Stop Hypertension diet.^[51,52]^ Furthermore, extra-virgin olive oil, a key component of the MD diet, can potentially explain the prevention of major CV events. Extra-virgin olive oil is rich in polyphenols, which help modulate endothelial function and reduce platelet activation,^[53]^ effectively preventing major CV events.^[54]^

Although our systematic review report MD superior efficacy over LFD in improving CV risk factors and outcomes, several limitations should be considered when interpreting the findings. First, the included randomized controlled trials exhibited heterogeneity in their design, particularly in the specific composition of the MD and LFD interventions. Second, the duration of follow-up across the included studies ranged considerably, from as short as 3 months to as long as 7 years. This variability in follow-up duration may have influenced the observed outcomes, particularly for clinical events that often manifest over extended periods. Studies with shorter follow-up may have been more sensitive to changes in surrogate markers like lipid profiles and inflammatory markers. In contrast, longer-term studies provided more insight into clinical outcomes. Third, several included studies assessed dietary adherence primarily using self-reported measures, susceptible to recall bias and social desirability bias, potentially leading to overestimating adherence to the assigned dietary interventions. Fourthly, the number of studies reporting CV outcomes (myocardial infarction, stroke, cardiac death, etc) was limited to only 2 trials.^[39,40]^ Furthermore, the discrepancies exist between the 2 studies, potentially due to differences in study populations and specific implementation of the dietary interventions. Finally, the study populations in the included trials varied in terms of baseline CV risk, presence of existing CVD, and geographical location, limiting the generalizability of our findings to all populations and highlighting the need for further research in diverse clinical settings.

Regarding future research, future RCTs with a large sample size, sampling from diverse ethnic and geographical groups, and long-term follow-up are required to assess the CV outcomes further. Furthermore, these trials should strive for greater standardization in the definition and implementation of the MD and LFD interventions. These trials should also consider the cost-effectiveness and sustainability of these dietary interventions in real-world settings. Additionally, future research should investigate the underlying mechanisms through which the MD exerts its cardioprotective effects. Exploring the impact of specific dietary components and their interactions on various biological pathways, including inflammation, endothelial function, platelet aggregation, and the gut microbiome, could provide valuable insights into the observed benefits. Finally, comparative effectiveness research directly comparing the MD with other established cardioprotective dietary patterns beyond LFDs, such as the Dietary Approaches to Stop Hypertension diet or plant-based diets, would provide a more comprehensive understanding of optimal dietary strategies for CV health.

Consistent evidence supports the MD’s value for primary and secondary CVD prevention by improving risk factors.^[55]^ Healthcare providers should recommend and educate patients on its principles (fruits, vegetables, whole grains, legumes, nuts, olive oil, moderate fish/poultry, limited red/processed meat), recognizing its potential benefits over a sole focus on fat restriction. Especially in secondary prevention, the MD should be integrated into comprehensive management. Individualized counseling, addressing cultural and economic factors, and food access is vital for adherence. Public health should promote MD principles through education and policies that enhance access to healthy foods. While promising, MD recommendations should align with evidence-based guidelines and individual needs, pending further research on hard outcomes.

## 5. Conclusion

This systematic review of randomized controlled trials indicates that the Mediterranean diet (MD) generally demonstrates favorable effects on several CV risk factors, including lipid profiles, inflammatory markers, glucose metabolism, insulin sensitivity, blood pressure, and anthropometric indices, when compared to a low-fat diet (LFD). While emerging evidence from some included studies suggests a potential protective effect of MD against CV events, particularly in secondary prevention, the findings on hard outcomes are limited and show some discrepancies. Therefore, these results highlight the potential of the MD as a dietary intervention for improving CV risk factors, supporting its consideration as a component of strategies for CVD prevention. However, due to the inherent heterogeneity in study designs and the limited number of trials specifically assessing long-term CV outcomes, further robust, large-scale randomized controlled trials with diverse populations and standardized dietary interventions are warranted to provide more definitive conclusions and to explore underlying mechanisms and cost-effectiveness in real-world settings.

## Author contributions

**Conceptualization:** Maneeth Mylavarapu.

**Methodology:** Maneeth Mylavarapu, Aarushi Batra, Israel Garcia, Abirami Balasubramanian, Muhammad Ammar Husnain, Ragha Harshitha Atla, Poorna Sai Harshini Muttuluru, Elias Abboud, Alisson Silva.

**Supervision:** Maneeth Mylavarapu.

**Visualization:** Maneeth Mylavarapu.

**Writing – original draft:** Maneeth Mylavarapu, Aarushi Batra, Israel Garcia, Abirami Balasubramanian, Muhammad Ammar Husnain, Ragha Harshitha Atla, Poorna Sai Harshini Muttuluru, Elias Abboud, Alisson Silva.

**Writing – review & editing:** Maneeth Mylavarapu, Aarushi Batra, Israel Garcia, Abirami Balasubramanian, Muhammad Ammar Husnain, Ragha Harshitha Atla, Poorna Sai Harshini Muttuluru, Elias Abboud, Alisson Silva.

## Supplementary Material
