# Supplementary material for: Mediterranean diet versus low-fat diet on cardiovascular disease (CVD) risk factors and outcomes: A systematic review of RCTs
Source: Medicine (Baltimore). 2026 Mar 13;105(11):e47971. doi: 10.1097/MD.0000000000047971 (PMC12991705; doi:10.1097/MD.0000000000047971)
Supplement: Supplementary file 1 [file medi-105-e47971-s001.docx]

**Supplement Table 1. Search Strategy**

| **PubMed/MEDLINE:**  ("Mediterranean Diet"[tiab] AND "Low-Fat Diet"[tiab]) AND ("Cardiovascular Diseases"[Mesh] OR "Heart Diseases"[Mesh] OR "Myocardial Infarction"[Mesh] OR "Stroke"[Mesh] OR "Cardiovascular Risk Factors"[Mesh])  *Filters: Randomized Control Trials* | 59 |
| --- | --- |
| **SCOPUS:**  "Mediterranean Diet" AND "Low-Fat Diet" AND ( "Cardiovascular Diseases" OR "Heart Diseases" OR "Myocardial Infarction" [mesh] OR "Stroke" OR "Cardiovascular Risk Factors" ) | 79 |
| **Science Direct:**  "Mediterranean Diet" AND "Low-Fat Diet" AND ( "Cardiovascular Diseases" OR "Heart Diseases" OR "Myocardial Infarction" [mesh] OR "Stroke" OR "Cardiovascular Risk Factors" )  *Filters: Research Articles; English* | 630 |
| **Clinicaltrials.gov:**  Mediterranean Diet; Low Fat Diet; Cardiovascular Disease | 14 |

**Supplement Table 2. Inclusion & Exclusion Criteria**

| **Inclusion Criteria:** |
| --- |
| 1. Randomized controlled trials (RCTs) comparing the Mediterranean diet and low-fat diet. |
| 1. Studies reporting cardiovascular disease risk factors (e.g., blood pressure, lipid profiles, inflammation markers) as outcomes. |
| 1. Studies report the incidence of cardiovascular events (e.g., myocardial infarction, stroke, cardiovascular mortality) as outcomes. |
| 1. Studies published in English. |
| 1. Studies with available full-text articles. |
| **Exclusion Criteria:** |
| 1. Non-randomized studies. |
| 1. Studies do not compare the Mediterranean diet and the low-fat diet. |
| 1. Studies with inadequate data reporting. |
| 1. Non-English studies. |
| 1. Studies lacking full-text availability. |
